# MiniVStimA: A miniaturized easy to use implantable electrical stimulator for small laboratory animals

**DOI:** 10.1371/journal.pone.0241638

**Published:** 2020-10-30

**Authors:** Manfred Bijak, Martin Schmoll, Jonathan C. Jarvis, Ewald Unger, Hermann Lanmüller

**Affiliations:** 1 Center for Medical Physics and Biomedical Engineering, Medical University of Vienna, Vienna, Austria; 2 Faculty of Science, John Moores University, Liverpool, United Kingdom; University of Maryland Baltimore County, UNITED STATES

## Abstract

According to PubMed, roughly 10% of the annually added publications are describing findings from the small animal model (mice and rats), including investigations in the field of muscle physiology and training. A subset of this research requires neural stimulation with flexible adjustments of stimulation parameters, highlighting the need for reliable implantable electrical stimulators, small enough (~1 cm^3^), that even mice can tolerate them without impairing their movement. The MiniVStimA is a battery-powered implant for nerve stimulation with an outer diameter of 15 mm and an encapsulated volume of 1.2 cm^3^ in its smallest variation. It can be pre-programmed according to the experimental protocol and controlled after implantation with a magnet. It delivers constant current charge-balanced monophasic rectangular pulses up to 2 mA and 1 ms phase width (1 kΩ load). The circuitry is optimized for small volume and energy efficiency. Due to the variation of the internal oscillator (31 kHz ± 10%), calibration measures must be implemented during the manufacturing process, which can reduce the deviation of the frequency related parameters down to ± 1%. The expected lifetime of the smaller (larger) version is 100 (480) days for stimulation with 7 Hz all day and 10 (48) days for stimulation with 100 Hz. Devices with complex stimulation patterns for nerve stimulation have been successfully used in two in-vivo studies, lasting up to nine weeks. The implant worked fully self-contained while the animal stayed in its familiar environment. External components are not required during the entire time.

## Introduction

Basic *in-vivo* research is often carried out in animal models because such experiments can be carefully controlled and reproduced, are scalable, and allow for detailed analysis of even rare pathologic conditions. In the years 2012–2018, an average of 1.2 million publications were added annually to PubMed [1]. About 720.000 of these papers were related to humans (search term: "year"[PDAT] AND "humans"[MeSH Terms]) and 226.000 to animals (search term: "year"[PDAT] AND "animals"[MeSH Terms:noexp]). Half of the animal-related papers used small rodents (mouse and/or rat).

Investigations in the field of muscle physiology and muscle training often observe changes induced by specific patterns of contraction. Instead of relying on relatively unpredictable voluntary muscle activation, it can be beneficial to have artificial motor control. This can be achieved via electrical stimulation of either nervous [2] or muscular tissue [3, 4], resulting in a need for a flexible and fail-safe stimulation system. While external systems might be used during acute experiments, long-term electrical stimulation can only be conducted reliably with implanted pulse generators (IPG). In the past, simple devices were used to produce a series of constant pulses at a fixed frequency [5, 6]. Ongoing progress in modern micro-electronics enables more sophisticated devices, which leads to increases in functionality and lifetime. Modern IPG's further allow for autonomous execution of even complex patterns of daily stimulation [7].

When interested in investigating particular properties in the living subject, a certain degree of biological variation must be taken into account. Usually, a high number of subjects, a control group, and a reproducible study design are required to consolidate conclusions statistically. The testing of different hypotheses (e.g., various combinations of stimulation parameters, different duty cycles, etc.) raises the number of individual experiments even further, resulting in a need for devices that are not only functional but also affordable. The use of IPGs allows programmed exercise to be delivered to, for example, one hind limb and not to the other. Therefore, each animal acts as its own control. In contrast, in experiments relying on whole-body exercises such as treadmill running, separate groups of animals are needed to provide control data (e.g., [8]).

Experiments to induce muscular adaptations employing resistance training last typically for about 6 to 36 weeks [9–14]. While the training is usually accomplished by conducting specific weight-lifting exercises [10–13] or by external electrical nerve stimulation of the anesthetized animal [9, 14], implantable devices rely on a portable power source. In battery-powered implants, the overall volume, as well as the lifetime, is mainly determined by the capacity of the battery used. The final design is, therefore, always a compromise between size and lifetime.

Several valuable publications from basic research exist, describing circuitry (ASIC) development for specific applications and proofed their functionality on the benchtop or in acute experiments, but did not find their way to long-term use in the animal (examples [15–22]). Since it is a long way from the workbench to a long-term-stable *in-vivo* implantable pulse generator, they are not further introduced.

In the past, an implantable heart pacemaker for mice [4] was used to deliver a continuous train of pulses at a fixed frequency (one pulse per heartbeat). It allowed selecting from four different pacing rates, which could be changed via an external magnet. This rather simple device, which measured 12 mm in diameter and had a mass of 1.5 g, was in use for up to 14 days. The authors stated that the constant-voltage (3-V lithium battery) output stage should be considered as the limiting component, as potential fibrosis or surrounding fluid might cause an electrical shortcut of the device, which is why they limited the reliable lifetime of the device to 7 days.

A more sophisticated device was developed for bilateral deep brain stimulation in the freely moving mouse [23]. The two-channel device delivered pre-programmed stimulation patterns with constant-stimulation pulses up to 100 μA, had a diameter of 8 mm, a length of 30 mm, resulting in a volume of 1.5 cm^3^ and a weight of 2.1 g. The increased functionality was achieved by a microcontroller, which limited the expected lifetime of the device to 10 h when active (15 days during standby) when using 3xSiO2 type 337 coin cell batteries.

A battery-powered device for deep brain stimulation but worn externally is described in [24]. The electronic components fit on a 30 mm x 14 mm circuit board and were packed with the batteries in a 3D printed plastic package, resulting in a device weighing 13.8 g. The authors concluded a battery life of 6 days and proposed a removable battery concept.

Kuazani et al. [25] targeted deep-brain stimulation with continuous stimulation current pulses of 200 μA and 90 μs phase width at 130 Hz. The device's circuit board was 15 mm x 18 mm in size and held a CR2032 battery (20 mm x 3.2 mm) with 235 mAh. They achieved a lifetime of 23 days.

Another approach to power the IPG is via an externally generated electromagnetic field, so-called radio-frequency (RF) powered IPGs [7, 26, 27]. The lifetime is considered infinite but only in terms of energy management. RF-powered systems require a transmitter coil in the vicinity of the animal, at least during periods of activity, leading to additional installations at the animal housing facilities. If only one implant can be addressed at the time, isolation of the animal during stimulation is required. Systems with addressable implants can theoretically stimulate multiple animals living in the same housing at the same time [7, 28].

Other approaches exist like tiny implants with rechargeable batteries [29] requiring frequent charging cycles or ultrasonically powered devices [19, 30] that relay on an ultrasonic transducer during stimulating. All of them need additional animal handling or efforts during the experimental time, or equipment installed to or near the animal housing.

Concerning animal welfare, it is desired to minimize the amount of additional stress caused by the intended procedure. One crucial criterion for implantable devices is, therefore, a small volume. Not only because the intracorporeal space is very limited, but also because it reduces the risk of rejection or skin abrasion. Further, a small system is essential to enable undisturbed movement and preservation of the general body image, which leads to a better acceptance of the implanted foreign body. When considering the increasing popularity of genetically modified mouse models to study specific diseases, the overall volume of an implant should not exceed 5 cm^3^ in rats. It must be even smaller (approx. 1 cm^3^) when targeting mice [31].

In addition to the requirements of small size, the full functionality of the nerve-stimulation-implant (monophasic, rectangular, charge-balanced pulses) must be easily accessible by implementing a self-contained design. This is essential for a reliable operation throughout the entire experimental period, leading to a minimized amount of manual interventions and keeping mistakes in the study protocol as low as possible.

The battery must last for the entire study period, implying a highly energy-efficient circuit design, having in mind that energy efficiency must be balanced with the accuracy of timing, speed, and computational power.

High reliability of operation and user-friendliness are the primary development goals of this device. All others are subordinated to these goals. The reliability of operation requires the device to accurately deliver the intended stimulation pattern without failures throughout the entire investigational period. User-friendliness means that all functions are easily accessible even for inexperienced users, that it is possible to start and stop the device after implantation at any time and that the user can freely choose between different predefined stimulation paradigms.

## Material and methods

The fully implantable pulse generator (IPG) was designed to be flexible and easy to handle, suitable for a great variety of research applications. The *MiniVStimA* requirements are based on previous experiences [31, 32] and act as a stand-alone system with pre-programmed firmware tailored to each specific application. They were intended for experimental studies that do not require regular or substantial modification of stimulation parameters during the experiment. Different stimulation patterns can be selected by bringing a permanent magnet near the implant, triggering a reset via an internal reed switch, and advancing to the next pre-programmed stimulation pattern.

### Functional description

**Fig 1:**
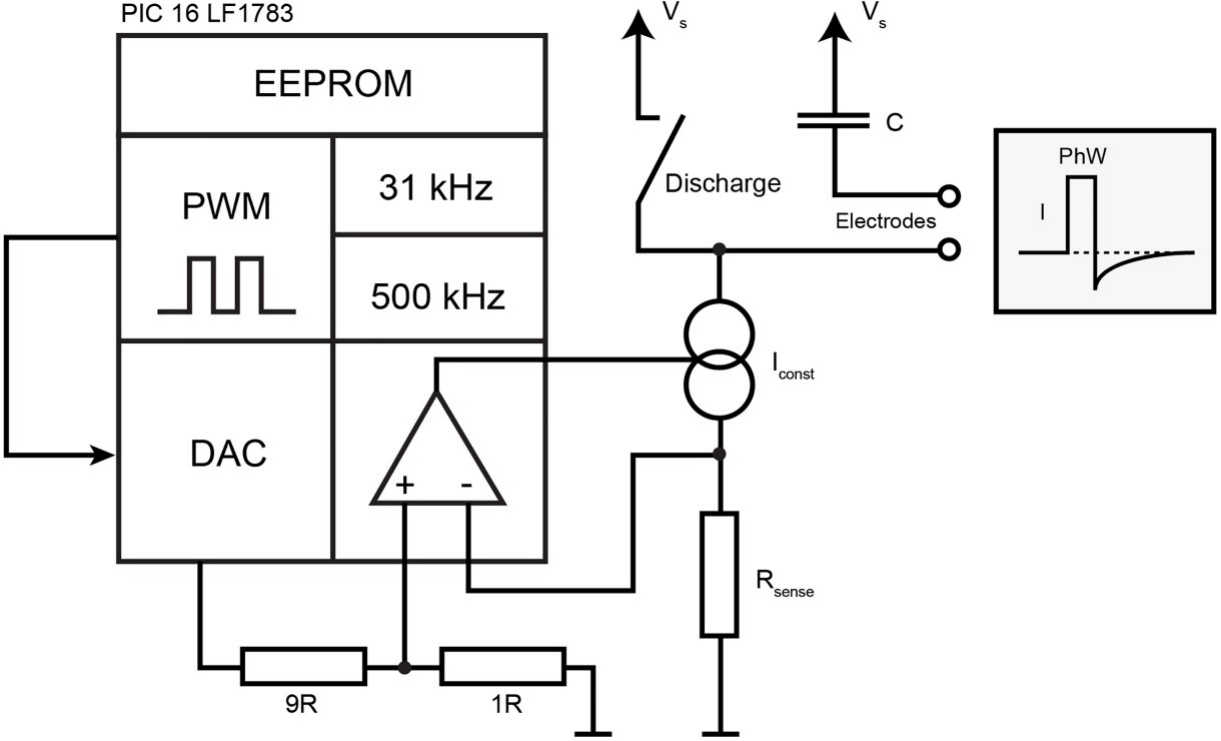
Functional blocks used to generate pulses. Data for the desired stimulation pattern was stored in the EEPROM of the microcontroller. The microprocessor's pulse-width-modulation (PWM) module was used to generate digital pulses that were forwarded to the digital-to-analog-converter (DAC) module. The output of the DAC module was presented to the operational-amplifier, which controlled a bipolar transistor to realize a constant-current-source (Iconst). Current delivered to the tissue charged a capacitor (C), which allowed to generate a charge-balancing second phase by closing an additional discharge-switch after the pulse.

The entire circuitry is built around the microcontroller PIC16LF1783 (Microchip, Chandler, AZ, USA), integrating a pulse width modulation (PWM) module, a digital to analog converter (DAC) and an operational amplifier (OPA), as shown schematically in Fig 1.

The integrated PWM module is used to generate rectangular constant voltage pulses with 0 V as low level and supply voltage (V_s_) as high level. As the module operates independently from the running firmware, it did not require additional software resources. It uses the same clock frequency (31 kHz), leading to a minimum phase width of one clock cycle, 32 μs respectively. By setting the internal registers of the PWM module, a wide range of phase width and frequency combinations can be realized.

The pulse amplitude is set by connecting the PWM output to the reference voltage input of the integrated DAC and down-regulated from the battery voltage by its resistor network. The DAC output voltage is further divided by a factor of 10 to control a constant current source (I_const_) formed by the integrated OPA and a bipolar transistor. R_sense_ provides feedback for the OPA allowing for the exact adjustment of the output current. The collector pin of the transistor is connected to one stimulation electrode while the other electrode is coupled via a capacitor to the supply voltage. The 1μF capacitor C (Fig 1) blocks direct current components and is a necessary safety feature to avoid DC induced electrode dissolution or tissue damage [33].

During constant-current pulse delivery C is charged, its voltage drop rises linearly over time. After the pulse ends, the switch labeled "Discharge" is closed, C is discharged. The switch is realized by a microprocessor output pin that is set High (= V_s_, low impedance). During pulse delivery, the pin is in tri-state mode (high impedance).

The described circuit is powered by either a standard 3 V lithium CR1220 battery (35 mAh) in the MiniVStimA35, alternatively with a DL1/3N battery (170 mAh) in the MiniVStimA170. A reed switch is used to disconnect all electrical components from the battery via a MOSFET, also enabling a power-on hard-reset of the microcontroller. A debouncing circuit takes care that the microprocessor resets only once during attaching/detaching of an external magnet.

### Circuit design

To achieve the primary goal, "high reliability of operation", the number of components and contact points are minimized. Moisture ingress is a known problem in implants without hermetically sealed metal or ceramic cases. Consequently, distances between contacts are kept as high as possible, keeping a reasonable compromise to implant size, which also implies not always selecting the electronic components in their smallest available casing.

**Fig 2:**
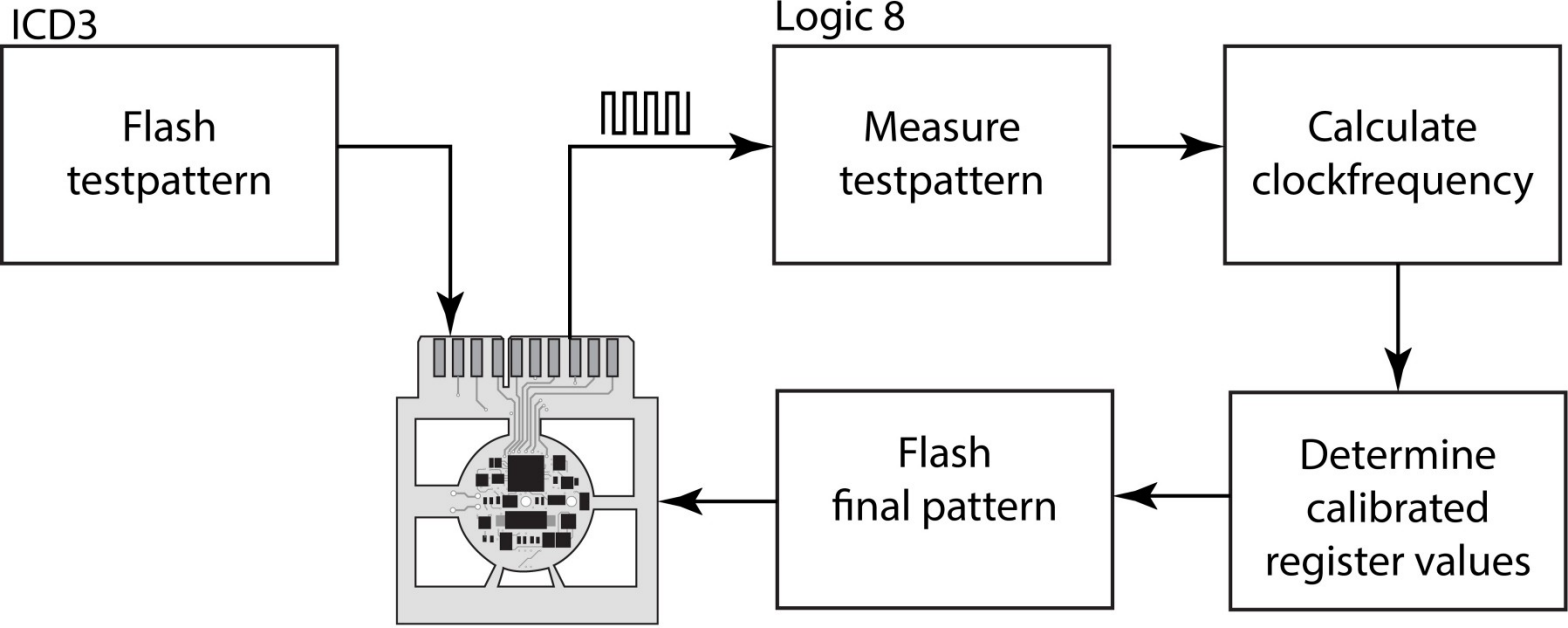
Temporal calibration process. An automatic calibration process is integrated when preparing the devices. A test pattern producing a well-defined periodic output signal is flashed to the microprocessor of the implant. From the actual clock frequency, calibrated register values are determined for the final pattern, compiled, and flashed in the last step.

The entire circuitry consists of the microprocessor (case SSOP28), 8 resistors (case 0603), 3 capacitors (case 0603), one bipolar transistor (PUMX1, case SOT363), 3 MOSFET (FDG6320, case SOT-323-6) and one reed switch. The microprocessor is mounted on one side of the 12 mm wide circular 2 layer board, and all the other components are placed on the opposite side. For better handling and automatic pick-and-place, the implant is surrounded by a PCB frame (Fig 2). The PCB frame contains a connector for in-circuit flashing of the microprocessor, some testing points, two connections for the battery, and two connections for the electrode leads.

After successful flashing and testing, the 12 mm implant disc is carefully cut out of the outer frame and prepared for encapsulation.

### Energy-saving measures

The size of the battery is one option to increase implant lifetime; an additional one is to optimize the circuitry in terms of energy efficiency, carefully aiming for lower current consumption, especially between stimulation pulses.

The selected microcontroller is designed for low-power applications offering a variety of power-saving features. Relevant is the usage of the internal low-power RC oscillator with its nominal frequency of 31 kHz. The internal 500 kHz oscillator has higher accuracy (±1%) but is only used for short periods when higher computational power is required, mostly when writing to or reading from the internal EEPROM.

It is also essential to consider the high current consumption of the OPA, ranging from 300 μA to 700 μA (@ 3V supply voltage, value not guaranteed by the manufacturer). Fortunately, the OPA (and DAC) must only be active during pulse generation and can stay off for most of the time—even between consecutive pulses. Although the OPA only requires 10 μs to settle after activation, it must be switched programmatically (1 instruction = 4 clock cycles) while the PWM module for pulse generation is running independently. The PWM related timer-register must be polled continuously to activate the OPA and DAC right before the pulse is delivered. Applying a certain safety margin concerning the compiler optimizations and possible interrupts requires switching on OPA and DAC 2 ms before pulse delivery, resulting in a maximal stimulation frequency of 100 Hz, where OPA and DAC can be efficiently switched off between pulses (PWM mode 1). For higher stimulation frequencies, the OPA and DAC must stay active while pulses are delivered (PWM mode 2).

The microcontroller's watch-dog timer (WDT) is usually used to reset the device and offers the possibility to continue the program after waking up from a low-power sleep-mode. The sleep-mode, with its current consumption of only 50 nA, makes it possible to realize energy-efficient rest times between consecutive stimulation bursts.

The WDT may also be used at low frequencies to sleep the microcontroller between pulses. The latter implies that the stimulation pulse has to be generated programmatically (not with PWM), limiting the phase width and interphase intervals to integral multiples of 129 μs. In this case, the OPA and DAC can be switched one program cycle before and after the pulse without safety margins ("firmware controlled pulse generation").

### Temporal calibration

Preliminary measurements performed on 88 microprocessors at room temperature (25°C) revealed that the nominal clock-frequency of 31 kHz varies within a range of 28.6 kHz to 33.4 kHz. (Tests at an intracorporal temperature of 37°C demonstrated, that the clock-frequency changes due to the temperature difference are neglectable). It could be argued that this deviation would only have a minor influence on the selected stimulation parameters, e.g., a nominal 129 μs pulse could be expected between 119 μs and 139 μs, and a nominal stimulation frequency of 50 Hz might be in the range of 46 Hz to 54 Hz. This inaccuracy can be accepted from a physiological point of view, but it would cause substantial problems when it comes to long-term applications. A stimulation pattern that is active for only 15 min once a day would restart every 22.1 h to 25.9 h instead of the desired 24 h. In *in-vivo* experiments, it can be required to check the stimulation at regular intervals, which can be challenging considering these temporal variations.

Therefore, each device is calibrated individually (Fig 2) before use. This calibration process is integrated into the final automated implant testing procedure. Before the circuit board is cut out of its frame, it is connected to the programming device (ICD3, Microchip, Chandler, AZ, USA) and a logic analyzer (Logic 8, Saleae, San Francisco, CA, USA). Both manufacturers, Microchip and Saleae, provide a Dynamic Linked Library (DLL) to control their devices via function call from within Microsoft Visual Studio.net Development Environment.

In Microsoft Visual Studio, an application was designed, which first flashes the microprocessor with a simple program, to produce an alternating rectangular signal on an output pin. The logic analyzer measures the signal frequency, with which the exact RC oscillator frequency can be calculated. An optimization algorithm finds microprocessor register configurations for either the least error of the stimulation parameters or the longest microprocessor sleeping time for most energy-saving. The new parameter set is compiled along with the final firmware and automatically flashed into the device.

The entire process is documented in a Web-Based MySQL-Database for quality assurance.

### Implant encapsulation

**Fig 3:**
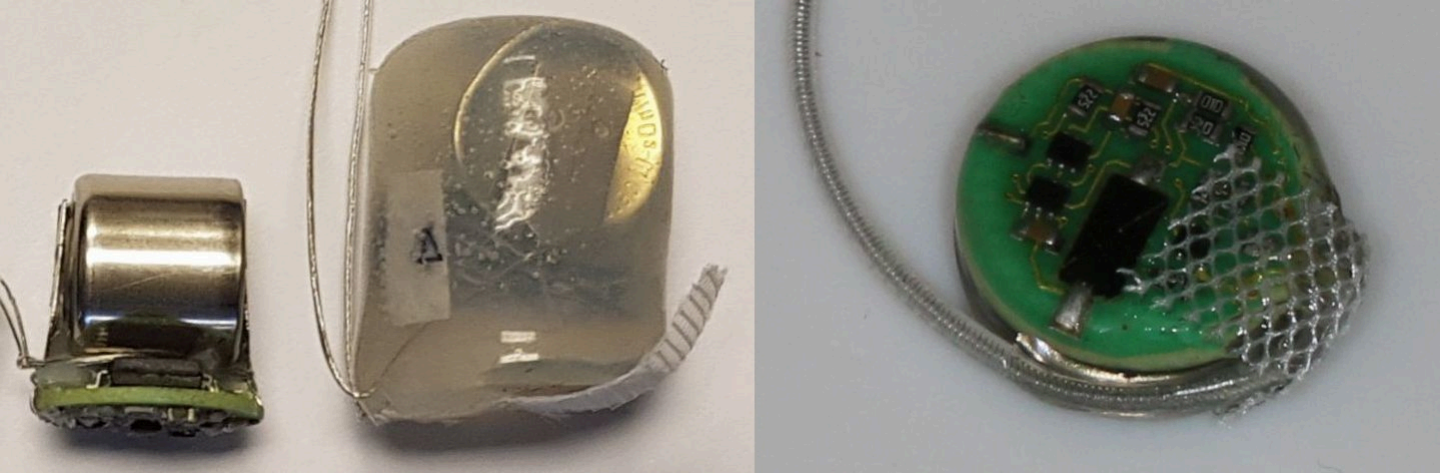
**Left: MiniVStimA170 (DL1/3N battery).** Middle: MiniVStimA170 encapsulated in silicone. Right: MiniVStimA35 (CR1220 battery) encapsulated in Expoxy Resin with attached electrodes.

Most of the manufactured implants were encapsulated in silicone rubber, using the technique described in [34] (Fig 3 / left, middle, MiniVStimA170).

To decrease the implant size to a minimum, the circuitry, together with the battery, could also be cast in medical-grade epoxy resin using a two-part casting mold. A loop of Dacron mesh, attached to the wall of the stimulator with medical-grade silicone adhesive, provided anchorage during implantation (Fig 3 / right).

### Quality management

For the initial function check, a firmware is flashed into the device, producing three basic patterns. In the first pattern, all microprocessor ports are set to their default values, followed by the sleep-mode. The second pattern generates pulses at 7 Hz / 258 μs / 2 mA with "firmware controlled pulse generation", followed by the third pattern with 100 Hz / 129 μs / 2 mA in "PWM mode 1". The output is loaded with a 1 kΩ resistor.

For all three patterns, the mean supply current is automatically measured, and all results are stored in a MySQL database.

### In vivo usage

**Fig 4:**
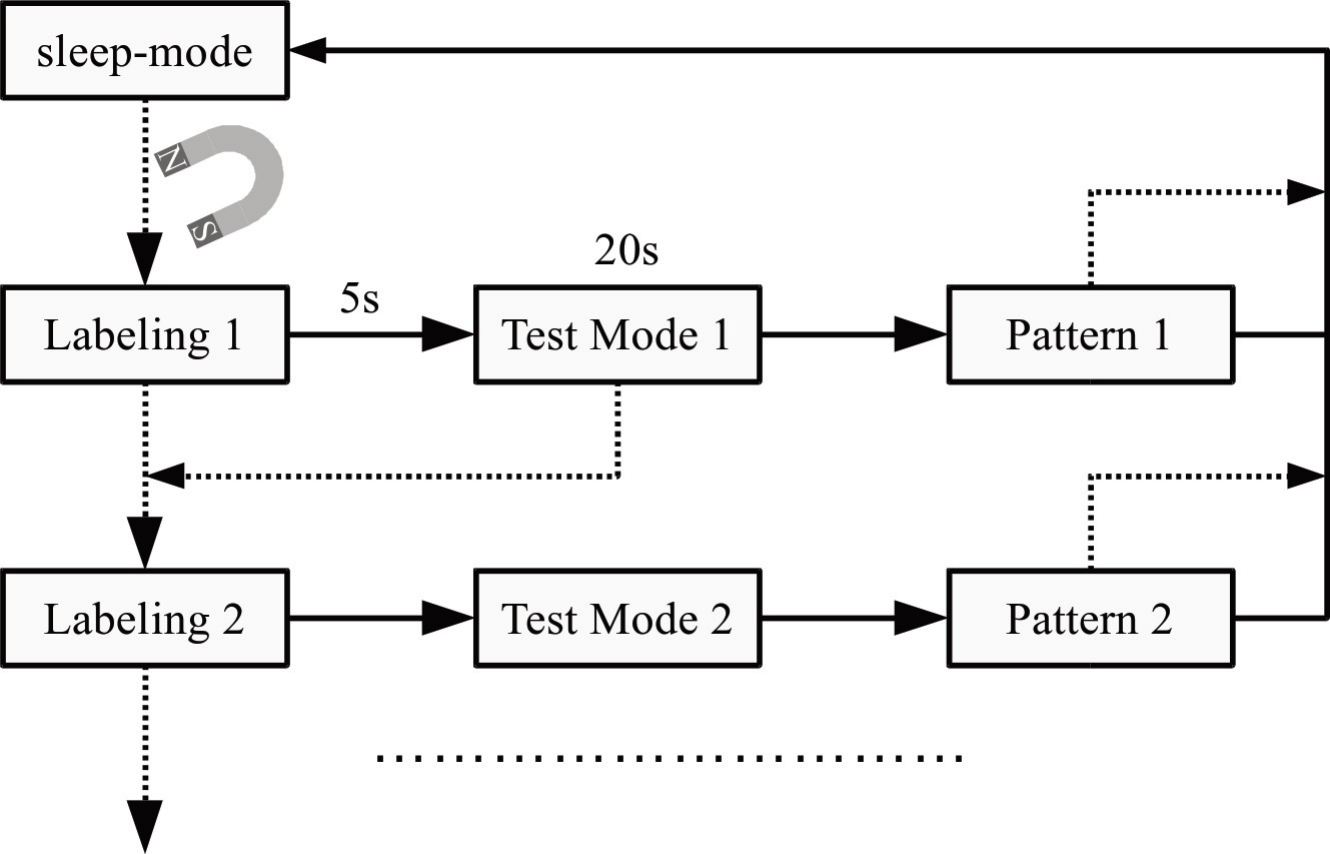
Implementing a labeling sequence followed by a test mode when more than one stimulation pattern is required. The dotted lines represent actions that are triggered, bringing a strong magnet near the implant.

For applications that require more than one stimulation paradigm, a "labeling" sequence to identify the chosen stimulation pattern, similar to a strategy used by Thiele et al. [35], was implemented and if needed, followed by a pattern test mode (Fig 4).

Labeling is done by delivering a related number of 1s spaced pulses with maximum stimulation amplitude, triggering recognizable reactions of the targeted tissue like muscle contraction. The after 5s started test mode delivers for 20 s pulses with the same stimulation parameters as the final stimulation pattern. That is useful to quickly check the stimulation during implantation since the following pattern does not necessarily start immediately stimulating.

The MiniVStimA was used in two published studies, [35] and [36]. All animal studies in [35] were approved by the Institutional Animal Care and Use Committee at the James J. Peters Veterans Affairs Medical Center and conformed to all guidelines and regulations for the protection of the welfare of animal subjects. All experiments in [36] received ethical approval and were carried out in strict accordance with the Animals (Scientific Procedures) Act of 1986.

## Results

240 MiniVStimA electronic circuitries were manufactured with PIC16F1783 (Silicone Revision B4), and 88 devices were assembled entirely (Table 1).

**Table 1:** Implant specifications of the MiniVStimA35 and MiniVStimA170.

	MiniVStimA35 (CR1220 battery)	MiniVStimA170 (DL1/3N battery)
Dimensions and weight		
Diameter	15 mm	15 mm
Height	7 mm	20 mm
Volume	1.2 cm^3^	3.5 cm^3^
Weight	2.3 g	7 g
Lifetime		
Expected lifetime (7 Hz continuous, 2 mA, 256 μs)	100 d	480 d
Expected lifetime (100 Hz continuous, 2 mA, 129 μs)	10 d	48 d
Expected lifetime in stand by mode	830 d	3900 d
Stimulation parameters		
Pulse shape	Monophasic, rectangular, charge-balanced	
Maximal current amplitude	2 mA	
Maximal voltage output	3 V	
Maximal phase width	1 ms	
Maximal stimulation frequency	200 Hz	

With the testing-firmware flashed, the microprocessor enters the sleep-mode shortly after removing the magnet. The related quiescent current is slightly higher, amounting to 1.7 ± 0.05 μA (range: 1.6 to 1.8 μA). With the energy-optimized 7 Hz pattern, an average current of 14.2 ± 0.3 μA (range: 13.5 to 15 μA) is drawn. The mean current (DC equivalent of the delivered pulses) through the load was, in this case, 3.6 μA (calculated for 2 mA output current, 7 Hz stimulation frequency, and 256 μs phase width).

With 100 Hz continuous stimulation, the current consumption was 145 ± 4.2 μA (range: 135 to 156 μA) with 26 μA going into the load.

**Fig 5:**
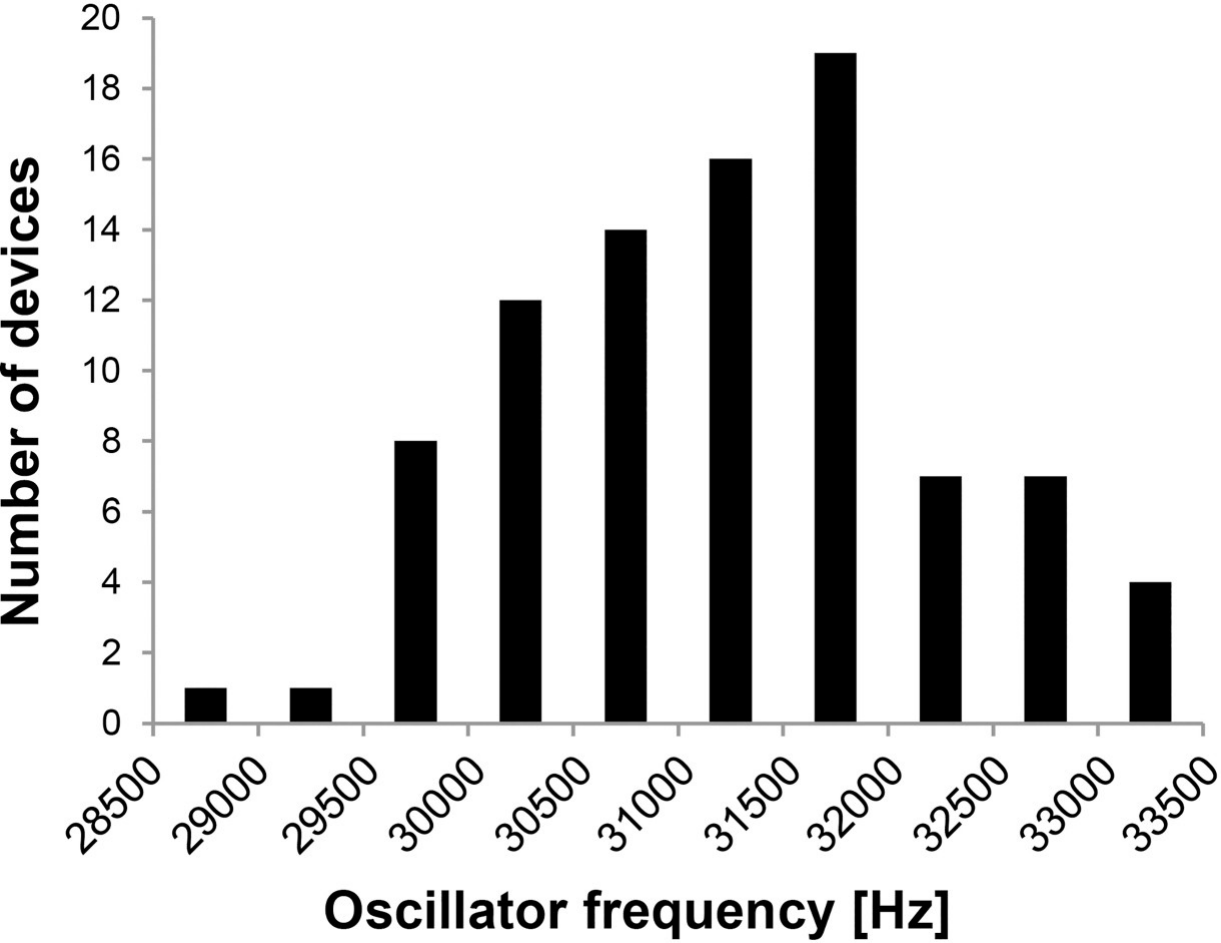
Distribution of clock frequency using the low power oscillator. The clock frequency of 88 devices using Microchip's PIC 16F1783 (Rev. 8) microprocessor was measured at room temperature.

The base for all time-related parameters is the oscillator frequency. Frequency measurement before flashing the final firmware revealed an RC oscillator frequency of 31270 ± 997 Hz, ranging from 28630 Hz up to 33350 Hz. The distribution of the oscillator frequencies when grouping them in 500 Hz ranges is summarized in the histogram of Fig 5.

A typical stimulation pattern that was used in 16 devices is outlined in Table 2. The pattern stimulated with 20 Hz / 387 μs for 10 s followed by 20 s rest for 12 h and then hourly for 10 s (3590 s resting time).

**Table 2:** Stimulation parameter ranges and deviation from the nominal value in% due to different oscillator frequencies.

	Nominal stim. parameter	Calculated stim. parameters due to f_Osc_ variation^3)^	Calculated stim. parameters with register values optimized	Measured stim. parameters from 16 devices^4)^	Time not in sleep
**Phase width [μs]**	387	428 to 353 (+10.7% to -8.8%)	428 to 353 (+10.7% to -8.8%)	421 to 360 (8.8% to -7%)	^5)^
**Stim-frequency [Hz]**	20	22.1 to 18.2 (+10.7% to -8.9%)	20.2 to 20.0 (+2.1% to 0.0%)	20.23 to 20.00 (1.2% to -0.0%)	^5)^
**Stim-On time [s]**	10	10.94 to 9.01 (+9.4% to +9.9%)	10.00 to 9.79 (0.0% to -2.1%)	9.95 to 9.83 (-0.5% to -1.7%)	^5)^
**Stim-Off time TMR0 [s]**	20	21.92 to 18.06 (+9.6% to -9.7%)	20.00 to 19.76 (0.0% to -1.2%)	^6)^	^5)^
**Stim-Off time WDT (5% error) [s]**	20	21.92 to 18.06 (+9.6% to -9.7%)	21.0 to 19.00 (+5.0% to -5.0%)	20.9 to 19.4 (+4.5% to -3.1%)	39 ms to 10 ms
**Stim-Off time WDT (1% error) [s]**	20		20.2 to 19.80 (+1.0% to -1.0%)	^6)^	308 ms to 10 ms
		3937 to 3242 (+9.7% to -9.7%)			
**Resting time [s]**^**1)**^	3590		3733 to 3447 (+4.0% to -4.0%)	3681 to 3448 (+2.5% to -4%)	26 ms to 28 ms
**Resting time [s]**^**2)**^	3590	3937 to 3242 (+9.7% to -9.7%)	3592 to 3588 (+0.05% to -0.06%)	^6)^	30 ms to 109 s

1) Stim. parameters optimized for minimal wakeup time allowing a maximal error of ±4%

2) Stim. parameters optimized for least deviation from nominal

3) Range of fOsc variation see Fig 5; register values calculated for fOsc = 31 kHz and constant

4) fOsc for the 16 devices varies between 28600 and 33400 Hz

5) Not applicable

6) This mode was not used / not programmed

For the related timing parameters, the possible value ranges were calculated by varying the oscillator frequency between 28 kHz and 34 kHz in 10 Hz steps, firstly with constant register values (calculated for the nominal oscillator frequency) and secondly with optimized register values (for each oscillator frequency). The measured values are taken from the 16 devices.

### Implant lifetime

The implant lifetime for a specific stimulation pattern can be estimated, according to (1).

$$D_{activ}=\frac{Q_{Batt}-{(D}_{standby}∙c_{sb})}{ppd∙(k_{1}∙I∙t_{P}+c_{opa})+c_{pb}∙bd+c_{sb}}$$ (1)

*D*_*activ*_ lifetime for a specific stimulation pattern (days)

*D*_*standby*_ number of days in standby mode since manufacturing

*Q*_*batt*_ battery capacity (mAh)

*c*_*sb*_ daily loss of battery capacity in standby mode (= 0.0014 mAh)

*ppd* stimulation pulses per day

*k*_*1*_ conversion factor pAs to mAh (= 0.28·10^−12^)

*I* stimulation amplitude (μA)

*t*_*P*_ stimulation pulse phase width (μs)

*c*_*opa*_ OPA caused battery drain per stim. Pulse (= 2.1·10^−7^ mAh)

*bd* burst duration of all bursts for one day (s)

*c*_*pb*_ battery drain during burst per second (= 1.2 · 10^−6^ mAh/s)

### In vivo applications

First, implants were used in a study to better understand the mechanisms of how muscle activity influences bone mass [36]. Five female Wistar rats were implanted to elicit near isometric contractions of soleus and plantaris muscles opposed by contractions of the tibialis anterior muscles.

Labeling was implemented according to Fig 4, with Pattern1 intended for intraoperative testing with continuous stimulation at 8 Hz, 128 μs, and 2 mA. Pattern2 provided stimulation over 8 days for 60 min daily, in a 2 s on and 18 s off regime at 40 Hz, 194 μs and 2 mA. No device failure was reported. The labeling strategy to identify selected patterns was well accepted.

Next, to investigate the "Morphological and histological adaptation of muscle and bone to loading induced by repetitive activation of muscle" [37] nine MiniVStimA were implanted in Wistar rats to induce contraction of the left tibialis anterior with 0.2 ms long pulses at 100 Hz for 200 ms every 30 s, resulting in a total of 9.6 min of stimulation per day. Stimulation was automatically delivered. The investigators did not report any device failure.

## Discussion

A fully implantable pulse generator intended for nerve stimulation with rectangular, monophasic charge-balanced pulses, MiniVStimA was developed, manufactured, tested, programmed according to the needs of different applications, encapsulated, and finally used *in-vivo*.

Several approaches to use ASICs [15–22] for implantable IPGs exist, but efforts and costs exceed the current project design. Microprocessors like the chosen PIC16F1783 are available in comparable small packages (e.g., UQFN case measuring 4x4x0.5 mm–which we did not use due to short pin distances), support "extremely low power management".

MiniVStimA35, with the 35 mAh CR1220 battery has a diameter of 15 mm and a height of 7 mm, resulting in a volume of 1.2 cm^3^ (Table 1), it is small enough to be used in the mouse model and, of course, larger animals (Fig 3). MiniVSimA170, with the 170 mAh battery DL1/3N mounted, has a volume of 3.5 cm^3^ (Table 1) and can be used in rats and larger animals. The usage of the DL1/3N battery extends the implant lifetime by a factor 4.8.

After manufacturing, the implant is switched (by the magnet) into standby mode. The current in this mode is 1.8 μA (mainly caused by the power-up circuitry), resulting in a standby time of 830 days (CR1220 battery) and 3900 days with the DL1/3N battery until the battery is fully drained (Table 1). The resulting discharge during storage reduces the active lifetime (when implanted). Since implants are produced on demand, the time from manufacturing to implantation is short and has practically no influence on the active lifetime. In comparison, Kuazani et al. [25] reported 425 μA in steady-state operation, and in [23], a standby time of 15 days could be achieved.

As soon as the IPG is touched again with the magnet, the first during manufacturing pre-programmed stimulation pattern starts. The lifetime after the IPG starts working depends strongly on the actual stimulation pattern and the amount of energy that is delivered to the tissue (stimulation amplitude, frequency, phase width), as expressed in (1). The primary parameter determining the lifetime is how much time the microprocessor can spend in sleep-mode with DAC, and OPA shut off. At low stimulation frequencies, sleep-mode can be activated even between pulses, resulting in an expected lifetime for an exemplary pattern with 7 Hz continuous stimulation at 2 mA / 256 μs of 100 days and 480°days, respectively for MiniVStimA35 and MiniVStimA170. At high stimulation frequencies, sleep-mode cannot be entered at any time, reducing the lifetime for 100 Hz continuous stimulation at 2 mA/256 μs to 10 days and 48days, respectively.

Since constant high-frequency stimulation causes muscle fatigue and further leads to muscle damage and necrosis, it is rarely used in this way. Typical is a short train of stimulation followed by a long rest time, allowing muscle recovery. This on-off regime prolongs the lifetime proportionally and must be calculated for each pattern accordingly to Eq (1). Additionally, the active lifetime depends on the chosen battery, and the measures to reduce current consumption like component selection, careful circuit design, and optimized microprocessor firmware, resulting in the active lifetimes listed in Table 1.

The three introduced studies [4, 23, 24], and [25] with similar implant dimensions reached lifetimes ranging from 10 hours up to 23 days.

An important strategy that we exploited to keep current consumption low is the usage of the internal 31 kHz RC oscillator. Moreover, the external quartz or ceramic resonator can be omitted, saving space and, as previous experiments showed, eliminate an error-prone component. The only drawback is the high tolerance (±10% from 31 kHz) of the oscillator (Fig 5). By contrast, the internal 500 kHz is calibrated to 1% deviation from nominal during manufacturing but requires roughly the tenfold supply current.

All time-related parameters are proportional to the oscillator frequency, and therefore have the same tolerance as the oscillator frequency. A ±10% deviation between devices might be acceptable in terms of stimulation frequency and phase width, but when conducting daily stimulation with the desire to check the animal during stimulation—a deviation of ±2.4 h within 24 h is not adequate.

The implemented calibration procedure (Fig 2) was able to improve the individual device accuracy significantly (Table 2). It decreased stimulation frequency errors from +10.7% to -8.9% down to +2.1% to 0.0%. The stimulation On-time (10 s) and the stimulation off-time (20 s), both generated with Timer 0, decrease down to 0% to -0.7% and +0.0% to -1.2%, respectively.

The stimulation off-time can also be generated with the watch-dog timer allowing the microprocessor to enter the sleep-mode. This measure requires wakeups in between to count the wakeup cycles for reaching a specific accuracy. Here we find some tradeoff, the more often the WDT wakes up, the higher the accuracy, but the more time not spent in sleep-mode, the higher the current consumption. In this example, the "time not in sleep" range can be decreased from 308 ms to 10 ms down to 39 ms to 10 ms if the allowed error range is increased from ±1% to ±5%.

Similar observations regarding the WDT supported sleeping were seen for the long term resting times (Table 2).

All stimulation parameter accuracies improved with calibration, except for phase width, which is directly derived from the clock frequency. A value of one in the related register means that the phase width is nominal 32 μs (1 / 31 kHz), a count of two 64 μs and so forth, leaving no space for optimization in the desired phase width range.

A drawback of the chosen implant concept is that it is difficult to adjust the stimulation intensity *in-vivo*. A solution was to pre-program several patterns with different amplitudes and used the introduced labeling strategy (Fig 4).

During usage, we learned that the labeling strategy worked well for up to 6 choices. The users reported that a higher number requires close attention to do the resetting/counting procedure. If the user is not very experienced, it takes two to four turns until the optimal intensity is fixed.

Since the stimulation parameters must be pre-programmed during the manufacturing process, the MiniVStimA is not intended for studies that require regular changes of the stimulation parameters, or where the stimulation parameters cannot be clearly defined beforehand. Other devices that are developed for higher flexibility must be used in these cases, like the BIONs [7].

On the other hand, the pre-programmed MiniVStimA does not require any technical expertise in setting it up and maintaining it. It is adjusted during implantation and does not require any additional devices (besides a magnet) during implantation. No other installations are necessary for the animal facilities, avoiding additional workload for the caretakers like disinfection after the end of the study and, according to the local protocol, probably repetitively.

## Conclusion and outlook

The MiniVStimA implantable pulse generator for nerve stimulation is with its 1.2 cm^3^ volume small enough for use in the mouse model, flexible enough to be adapted to various applications, simple to use, optimized for energy efficiency, and with both types of encapsulation, stable *in-vivo*.

Complex stimulation patterns can be pre-programmed and run fully self-contained over weeks while the animal stays in its familiar environment, an essential part of fulfilling user-friendliness.

Time-related stimulation parameters are proportional to the microprocessor clock frequency and vary between devices in a ±10% range. With a unique manufacturing procedure, the tolerance can be reduced to below ±1%, except pulse width.

Two *in-vivo* studies used the MiniVStimA without observing a single device failure, demonstrating the reliability of operation.

The use of a simple magnetic control-interface has the advantage that no other external programming equipment is required. The rodent can be held in one hand while palpating the targeted muscle. At the same time, the magnet can be manipulated with the other hand. Selection from a small number of preset programs is thus facilitated. We have found that for a more complex parameter setting, and simplified amplitude adjustment, a genuinely two-way data RF communication is desirable, which will be implemented in future designs.
